# Sensory-motor problems in Autism

**DOI:** 10.3389/fnint.2013.00051

**Published:** 2013-07-18

**Authors:** Caroline Whyatt, Cathy Craig

**Affiliations:** Department of Psychology, Queen's University BelfastBelfast, Antrim, UK

**Keywords:** autism spectrum disorders, perception-action coupling, prospective control, movement, developmental psychology

## Abstract

Despite being largely characterized as a social and cognitive disorder, strong evidence indicates the presence of significant sensory-motor problems in Autism Spectrum Disorder (ASD). This paper outlines our progression from initial, broad assessment using the Movement Assessment Battery for Children (M-ABC2) to subsequent targeted kinematic assessment. In particular, pronounced ASD impairment seen in the broad categories of manual dexterity and ball skills was found to be routed in specific difficulties on isolated tasks, which were translated into focused experimental assessment. Kinematic results from both subsequent studies highlight impaired use of perception-action coupling to guide, adapt and tailor movement to task demands, resulting in inflexible and rigid motor profiles. In particular difficulties with the use of temporal adaption are shown, with “hyperdexterity” witnessed in ballistic movement profiles, often at the cost of spatial accuracy and task performance. By linearly progressing from the use of a standardized assessment tool to targeted kinematic assessment, clear and defined links are drawn between measureable difficulties and underlying sensory-motor assessment. Results are specifically viewed in-light of perception-action coupling and its role in early infant development suggesting that rather than being “secondary” level impairment, sensory-motor problems may be fundamental in the progression of ASD. This logical and systematic process thus allows a further understanding into the potential root of observable motor problems in ASD; a vital step if underlying motor problems are to be considered a fundamental aspect of autism and allow a route of non-invasive preliminary diagnosis.

First identified in the seminal works of Leo Kanner (1943) and Hans Asperger (1944) Autism, also known as Autism Spectrum Disorder (ASD), is a developmental disorder characterized by impaired socialization, communication, and imagination (Wing and Gould, 1979; Wing, 1981; American Psychiatric Association, 2000). ASD research largely reflects this bias, with a strong focus on three core theories of Autism: Theory of Mind (Baron-Cohen et al., 1985), Weak Central Coherence theory (Frith, 1989), and Executive functioning theory (Ozonoff et al., 1991; Ozonoff and McEvoy, 1994).

This paper will provide a brief overview of these traditional theories, before outlining how research has attempted to profile and understand movement ability associated with a diagnosis of ASD. Combing specific examples, and discussing motor performance within the context of ecological psychology, we will draw well-defined links between standardized “norm” based assessment tools and in-depth kinematic movement analysis based studies. Specifically we will present sample studies that explore the role of timing and perception-action coupling in children with ASD who experience motor difficulties. These findings will then be discussed in light of the development of coherent movement control and its impact on social and cognitive ability, highlighting the potential role of a Theory of Sensory-motor control in ASD.

## Traditional theories of autism spectrum disorder

First coined by Premack and Woodruff (1978) “Theory of Mind” (ToM) refers to the ability to make inferences regarding others' intentions and emotions. Impaired ToM results in the inability to attribute separate mental states to individuals, leading to difficulty understanding and predicting others' feelings and behaviors; classical social symptoms of ASD (Baron-Cohen et al., 1985). Despite early criticism (e.g., Hobson, 1991; Russell, 1992) ToM has received strong support (e.g., Baron-Cohen et al., 1997) and is often regarded as the predominant theory in ASD research. However, upon closer inspection fundamental difficulties adopting this theory become apparent. Initial evidence alluded to a preserved level of ToM in some individuals with ASD (Baron-Cohen et al., 1985; Happe, 1995; Bowler, 2006), whilst ToM as a construct fails to reliably differentiate individuals with ASD from those with Down's syndrome, sensory impairment or intellectual disability (Baron-Cohen et al., 1985; Russell et al., 1998; Yirmiya et al., 1998). Deconstructing this concept further highlights the strong cognitive basis of ToM, thought to be largely dependent on the capacity for complex thinking and metarepresentation (Boucher, 2012), which are reliant on language based strategies. These strong links to language ability (Happe, 1995) raises the question, is ToM truly implicated in ASD, or, by using impaired language ability as a diagnostic criterion is this level of impairment naturally inflated?

*Weak Central Coherence theory* (Frith, 1989) provides an explanation for “non-social” symptoms of ASD such as apparent difficulties with global processing and preference for local level detail. Referred to as a cognitive style, weak central coherence results in difficulties considering contextual information leading to cognitive detachment. This predisposition to the minutiae of a scene is thought to result in superior performance on low-level visual tasks and illusions (Happe, 1996). Yet, conflicting results implying intact levels of global visual processing in ASD (Motton et al., 1999; Edgin and Pennington, 2005) undermine the reliability of this theoretical framework.

Finally, *executive functioning theory* aims to explain behavioral characteristics of ASD including rigidity in regime, spontaneous unreserved actions, and the need for order. Strongly interwoven with main constructs of ToM (Joseph and Tager-Flusberg, 2004; Pellicano, 2007), executive functioning is thought to provide a route of higher level control over automatic responses to stimuli, an ability to switch mind-set as required for example in the Wisconsin card sorting task, and to help formulate novel ideas (Frith, 2003). Despite evidence for reduced levels of executive function in ASD (e.g., Russell, 1997) this construct also fails to reliably differentiate between ASD and other disorders such as ADHD (Pennington and Ozonoff, 1996).

Combined these largely cognitive driven theories of ASD are functionalist and fragmented (see also De Jaegher, 2013), and fail to encompass the diverse range of symptoms associated with ASD. The strong cognitive thread throughout all “traditional theories” largely reflects the characteristic cognitive and social symptoms of ASD (American Psychiatric Association, 2000) yet is questionable given the ability of some individuals with ASD to reach high levels of academic success. In addition, the use of restricted language as a diagnostic criterion may lead to individuals with ASD displaying a predisposition for such higher-level cognitive difficulties (e.g., Lewis and Osbourne, 1990; Happe, 1995). Moreover these complex levels of cognitive functioning do not emerge until approximately 4 years of age in typically developing children (Wimmer and Perner, 1983; Perner et al., 1987; Harris et al., 1989; Boucher, 2012). As such, a purely cognitive explanation for ASD fails to account for autistic symptoms within the first years of an infant's life (Gillberg et al., 1990; Osterling and Dawson, 1994; Dawson et al., 2000).

When viewed in light of evidence that shows how cognition and motor ability develop in parallel and are mutually dependent (Campos et al., 2000; Von Hofsten, 2007; Rakison and Woodward, 2008; Iverson, 2010), a purely cognitive explanation of ASD is short sighted. Indeed, evidence for cognitive-motor links in ASD have already been documented by Hilton et al. (2007), who identified a strong correlation between motor impairment and level of severity of ASD as measured using the social responsiveness scale (Constantino et al., 2003). Coupled with evidence for the presence of significant sensory-motor problems in ASD from a very early age (Teitelbaum et al., 1998; Sutera et al., 2007), we propose that a fundamental, developmental sensory-motor deficit may be the missing link in understanding core elements of ASD.

Indeed, although predominantly viewed as a social and cognitive disorder, mounting evidence suggests the presence of significant sensory-motor deficits across the entire ASD spectrum (Manjiviona and Prior, 1995; Ghaziuddin and Butler, 1998; Jansiewicz et al., 2006; Fournier et al., 2010). However, in spite of this mounting evidence and early recognition of sensory-motor problems in ASD (e.g., Asperger, 1944; Damasio and Maurer, 1978; Vilensky et al., 1981), they remain to be seen as secondary, “associated” symptoms (Ming et al., 2007). A recent review (Fournier et al., 2010) suggested discrepancies in controlling for underlying moderating variables (e.g., IQ) along with the inclusion of control groups with secondary impairments (e.g., Developmental Coordination Disorder) could be preventing sensory-motor symptoms from being viewed as a core component of ASD. If sensory-motor problems are to be considered a fundamental symptom of ASD, the nature of persistent motor problems *specific* to ASD must be identified.

## Observable movement problems in ASD

Standardized tests of movement coordination are used by clinicians and researchers to assess the development of a broad range of motor skills. By comparing standardized scores, these tests are often the first step in identifying pronounced, observable motor deficits. A number of studies have used a range of standardized tests of motor performance to assess levels of motor proficiency in ASD (Manjiviona and Prior, 1995; Miyahara et al., 1997; Ghaziuddin and Butler, 1998; Green et al., 2002, 2009; Hilton et al., 2007; Provost et al., 2007; Staples and Reid, 2010; Siaperas et al., 2012). Although the number of research studies in this area is arguably limited, they provide preliminary evidence for persistent and significant observable motor difficulties across the Autistic Spectrum, with notable impairment in the sub-categories of manual dexterity and ball skills (Manjiviona and Prior, 1995; Miyahara et al., 1997; Green et al., 2002, 2009; Hilton et al., 2007). However, scoring methods commonly used in such standardized tests may inevitably mask underlying variation in performance. In particular, sub-category scores often rely on the summing of performance on multiple individual tasks. For example, performance in the sub-category of ‘*Ball Skills*’ in the Movement Assessment Battery for Children (M-ABC, Henderson and Sugden, 1992, 2nd Edition, Henderson and Sugden (2007)) relies on the summing of performance on two distinct tasks; a *‘Throwing’* and ‘*Catching*’ task (see Table 1). This is often further complicated by the scoring parameters included in individual tasks, with accuracy and speed used interchangeably (see Table 1).

**Table 1:**
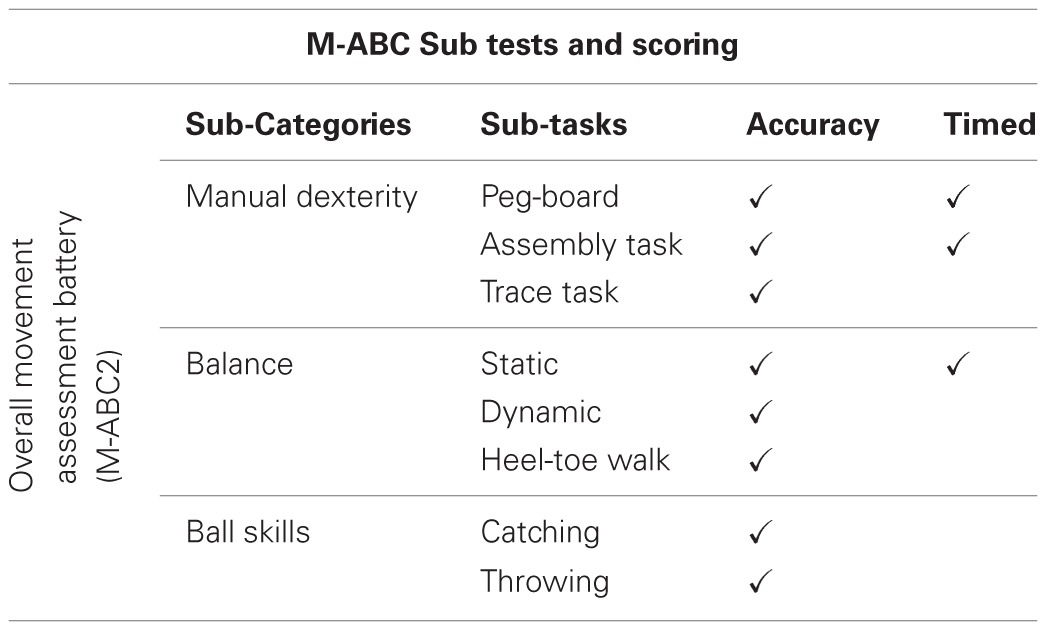
**Table outlining the construction of the movement assessment battery for children 2 (Henderson and Sugden, 2007)**.

*As shown, overall movement performance is assessed via the sub-categories; manual dexterity, balance and ball skills. Each sub-category is then further divided into performance on sub-tasks, each measuring individual levels of performance and scored according to either spatial accuracy and/or timed performance*.

To maximize the potential use of such standardized assessment batteries, we suggest deconstructing performance to consider ability at the individual task level, and viewing performance in light of differentiating factors (Whyatt and Craig, 2012). Comparing performance on the M-ABC2 (Henderson and Sugden, 2007), our recent study provided further evidence for persistent motor deficits in ASD in relation to age-matched children, with *no* secondary impairments (Whyatt and Craig, 2012). Moreover, supporting results from other studies, the breakdown of performance into specific sub-categories indicated the presence of significant difficulty in the areas of both *manual dexterity* and *ball skills* (Manjiviona and Prior, 1995; Miyahara et al., 1997; Green et al., 2002, 2009; Hilton et al., 2007; Provost et al., 2007; Staples and Reid, 2010; Siaperas et al., 2012). However, taking the deconstruction of performance to the individual task level revealed a specific pattern of impairment on a single task in each sub-category; *peg-board* task and *catching* task. Viewing the pattern of performance at this individual level, and in light of differentiating factors, suggests an underlying difficulty with the spatial-temporal control of movement. More specifically, catching requires the person catching the ball to prospectively control the movement of their catching hand as a function of the movement of the approaching ball. Therefore, performance on *catching* tasks is driven by externally imposed spatial and temporal constraints, where the dynamics of the moving object should guide the control of the action. Conversely, performance on the *throwing* task is predominantly internally driven, as the external contextual variables are stationary (i.e., no temporal constraints). Whilst questions are also raised over the reliability of the *peg-board* task, due to dual scoring using both spatial accuracy and age-related temporal parameters (see Table 1). Evidence for poor temporal awareness in ASD (Boucher, 2001) suggests this dual scoring component may artificially inflate levels of ASD impairment.

Moreover, given the body of evidence that suggests a significant relationship between IQ, specifically verbal ability (e.g., Leary and Hill, 1996; Chaix et al., 2007; Dziuk et al., 2007), and movement, both non-verbal and receptive language ability were independently controlled for (Whyatt and Craig, 2012). When these control group comparisons were carried out, further differences in ASD performance were noted. Overall impairment in the sub-category of *ball skills* and the underlying individual *catching* task was found in relation to both the non-verbal and receptive language control groups (*p* < 0.01). However, impaired levels of *manual dexterity* were seen to vary. Specifically, overall impairment in the sub-category was found when ASD performance was compared to the control group matched on receptive language ability only (*p* < 0.05). Yet underlying variation in performance on the individual *peg-board* task was isolated to comparisons with the non-verbal IQ control group (*p* < 0.05). This pattern of results highlights the difficulties encountered when using standardized tests, specifically their ability to reliably ‘mark’ variation, reinforcing the need to tease apart levels of performance, and implies a cognitive element to difficulties with *manual dexterity*.

Combined, these results may suggest a specific difficulty using external sensory information to prospectively guide and control action. However, despite this systematic deconstruction of performance, standardized product orientated tests still lack the sensitivity in measurement to unpick subtle variations in real-time patterns of performance.

## Internal vs. external timing: the role of perception action coupling

Internal timing, mediated by the basal ganglia (Graybiel et al., 1994; Gowen and Miall, 2005), is critical in the initiation of self-timed actions, for example reaching for a stationary object. However, despite being internally generated, unfolding temporal control over the movement will be directly modulated by external spatial parameters, for example as a function of target width (Fitts, 1954) or degree of curvature of the movement required (Viviani and Schneider, 1991). Conversely actions that require one to successfully couple movements onto that of the environment are driven and guided by externally imposed spatial and temporal constraints. For example, when catching a moving ball an individual needs to visually pick up information from the moving ball to anticipate where and when the ball will arrive and subsequently control the movement of the catching limb to arrive in the right place at the right time. Although largely taken for granted, this intricate relationship between the perception of the spatial and temporal characteristics of the moving ball and the control of the moving limb is critical to successful interception and is often described as *perception-action coupling*.

Information in the environment is thought to be continuously available from the eye in the form of the optic array (Gibson, 1969). Our movement through the environment then provides a time-varying optic array otherwise known as the optic flow field (Gibson, 1979; Lee, 1980) from which sensory invariants can be picked up and used to guide action (Gibson, 1969). These optical invariants are non-linear algorithms (Fajen, 2005), directly linking perception and action (Richardson, 2000) from which information can be extrapolated to provide prospective spatial and temporal control (Lishman and Lee, 1973; Lee, 1980). More specifically, research suggests that through maturity and perceptual attunement infants converge on the use of Time to contact information (Tau; Kayed and van der Meer, 2009) to allow them to prospectively control their movements. Tau in the visual domain is traditionally specified as the inverse of the rate of expansion of the image on the retina, whilst changes in the spectral and temporal characteristics of an auditory-based stimulus have also been shown to provide reliable time to contact information (Neuhoff and McBeath, 1996). Mathematically, tau is specified as the time to gap closure at its current closure rate (see Lee, 1980). In the example of catching an oncoming ball, Tau (τ) is calculated as the ratio between the distance gap separating the catcher and the ball (*x*) and the rate of closure (ẋ) of that gap so that:

(1) $$\tau (x)=x/\dot{x}$$

Extending this specification of temporal information further, other research has shown how the taus of two or more gaps can be closed synchronously to arrive at the right place at the right time (known as tau coupling—see Lee, 1998; Lee et al., 2001). Encompassing both temporal and spatial characteristics of the moving target, Tau provides reliable, robust information that the actor can tune into and use to successfully perform the task. Using tau-based information is therefore indicative of mature levels of prospective control. The gradual progression to this level of control would be evidenced in a person's ability to tailor the temporal characteristics of their movement, such as initiation time, to the event related information in the environment (e.g., the time to arrival of a moving target), resulting in higher levels of spatial/temporal accuracy of the movement and a reduction in the number of corrective sub movements (e.g., Von Hofsten, 1991; Van der Meer et al., 1994; Caljouw et al., 2004; Van Hof et al., 2008).

Studies that have examined movement kinematics in the ASD population have frequently documented pronounced difficulty with movement initiation (preparation), online control and smooth sequential actions (Hughes and Russell, 1993; Hughes, 1996; Rinehart et al., 2001; Mari et al., 2003; Schmitz et al., 2003; Glazebrook et al., 2006; Rinehart et al., 2006a; Cattaneo et al., 2007; Fabbri-Destro et al., 2009; Papadopoulos et al., 2012);. These difficulties emerge as an inability to prospectively control one's own movements (e.g., Hughes, 1996; Schmitz et al., 2003), but also a deficit in anticipating outcomes of others actions (e.g., Cattaneo et al., 2007). These underlying problems appear to reside in fundamental problems with the temporal control of movement, with both akinesia and hyperdexterity also being documented (e.g., Muller et al., 2001; Mari et al., 2003; Kleinhans et al., 2005; Rinehart et al., 2006a; Price et al., 2012a,b). This variability in movement timing is further significantly correlated with poor motor coordination (Price et al., 2012b), implying that spatial movement difficulties in ASD are in fact rooted in a more fundamental temporal deficit. In addition recent qualitative first hand reports provide rich evidence for temporal underpinnings, with reported difficulties “controlling movements,” “problems with starting or stopping movements,” and a tendency to “lose the rhythm” (Robledo et al., 2012. p. 6). Despite this, results are often attributed to an underlying difficulty with *motor programming;* specifically motor programme selection, re-programming and degradation (e.g., Rinehart et al., 2001, 2006a,b; Mari et al., 2003; Glazebrook et al., 2006, 2008; Nazarali et al., 2009). This implied motor programming deficit draws an explicit link between ASD and Parkinson's disease (PD), with distinguishing characteristics of PD such as akinesia and bradykinesia long considered the by-product of “an inability to select and/or maintain *internal* control over the algorithms” needed to generate actions (Robertson and Flowers, 1990, p. 591). This is of particular interest given recent evidence of patients with PD using external sensory information to improve the synchronization and timing of movements (Majsak et al., 1998, 2008). Comparing performance on a reach-to grasp task with a stationary and moving ball, Majsak et al. (1998, 2008) demonstrated how a dynamic moving target can act as an external ‘cue’ to time movement. By exploiting the perception-action link, the dynamic target provides *external* temporal information, which removes the emphasis on using *internal* temporal processes. The use of external temporal information therefore allows patients with PD to successfully overcome akinesia and bradykinesia to produce smooth sequential actions, implying a common underlying timing mechanism (Majsak et al., 1998, 2008). Given repeated evidence for a potential link between ASD and PD (Damasio and Maurer, 1978; Vilensky et al., 1981; Mari et al., 2003; Rinehart et al., 2006a; Vernazza-Martin et al., 2005; Hollander et al., 2009) such results highlight the potential importance of explicitly assessing levels of perception-action coupling in individuals with ASD.

Unfortunately, sensory-motor tasks used in ASD research to date are largely abstract, requiring mental retention and/or rotation to predict task outcome, which may artificially lower ASD performance (e.g., Leekman and Perner, 1991). Further, as noted by Van der Weel et al. (1996) goal-directed, concrete tasks which are controlled in such a way that sensory information (e.g., visual and auditory) is picked up from the environment and used to achieve the desired goal, are “true” sensory-motor tasks. Therefore, these abstract tasks fail to provide a true sensory-motor assessment and prevent results from being easily viewed within the context of observable motor problems such as those seen with standardized tests. To further unpick the potential role of external environmental constraints, namely sensory information on ASD temporal control, previous results (Whyatt and Craig, 2012) were used as a basis to design two targeted experimental paradigms which aimed to understand performance on a manual dexterity and interceptive task, in a more systematic way.

## Perception-action coupling studies

### Manual dexterity study (sample)

Manual dexterity refers to fine motor control of the small muscles in the hands and fingers to adequately manipulate objects and produce skillful performance. Although standardized testing has repeatedly implied poor levels of manual dexterity in ASD (Miyahara et al., 1997; Green et al., 2002, 2009; Hilton et al., 2007; Provost et al., 2007; Staples and Reid, 2010; Whyatt and Craig, 2012; Siaperas et al., 2012), recent evidence suggests this impairment is based on *specific* tasks scored using both *time* and *accuracy* parameters (e.g., peg-board), raising questions over the validity and reliability of this impairment (Whyatt and Craig, 2012). In particular, inherent variability in temporal production (e.g., Price et al., 2012b) and awareness (Boucher, 2001) may underpin poor performance on such dual-scored tasks.

**Figure 1:**
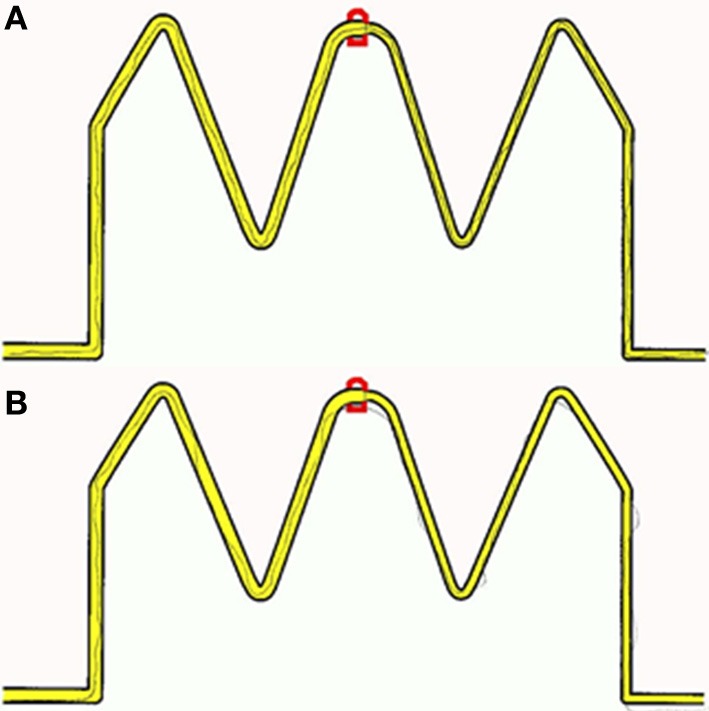
**Example of recorded traces produced by (A) a Non-verbal control participant (B) an age matched autistic participant**.

To provide participants with a controlled manual dexterity task, the original trace task from the M-ABC2 was digitized and presented on a tablet PC (see Figure 1 for example trace recordings). Performance was recorded with real-time visual feedback on the position of the line participants were drawing being instantly provided. Although not identified as a key task from the M-ABC2 (Whyatt and Craig, 2012), this task requires high levels of precision and perception-action coupling to prospectively control the movement to accurately navigate the pen between the boundaries of the drawing. Therefore, this task provides a strong test of fine motor control, yet is scored using accuracy parameters only. By digitizing the stimulus, sequentially deconstructing performance and viewing this in light of perceptual information (i.e., perceived width of tracks), a fuller understanding of true spatial-temporal control during fine motor tasks is achievable. Despite being internally generated, unfolding temporal control as the movement progresses will be directly modulated by external spatial parameters, for example target width (Fitts, 1954) or degree of curvature of the movement required (Viviani and Schneider, 1991). One would therefore expect high levels of spatial accuracy to be reflected in high levels of temporal or prospective control, for example an ability to prospectively control line drawing movement to avoid errors such as sufficient deceleration when approaching the corner sections. Data were filtered offline, from which displacement and temporal information were calculated. As before performance was compared between a group of children with a formal diagnosis of ASD and two age-matched control groups of typically developing children (non-verbal IQ and receptive language).

**Figure 2:**
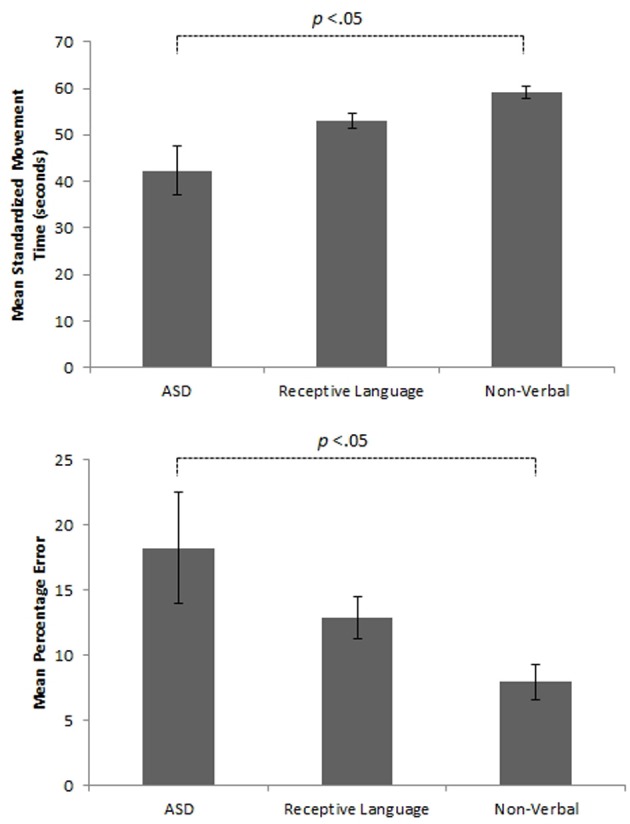
**Example summary graphs showing mean level of overall percentage error and associated standardized times (seconds) for the three different experimental groups**.

**Figure 3:**
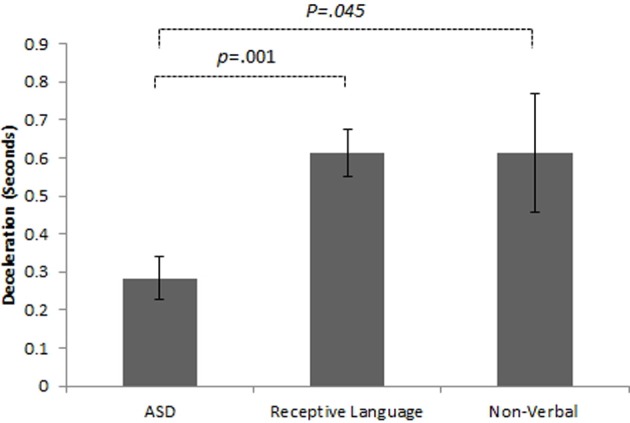
**Example summary graph for deceleration patterns when approaching a *single* comer section of the track task**. Combined analysis of performance on *all* comer sections highlights significantly shorter phases of deceleration in the ASD group than both the receptive language (*p* < 0.05) and non-verbal (*p* < 01) *control groups*.

Initial results of spatial accuracy imply significant ASD impairment throughout the task. However, in line with previous results (Whyatt and Craig, 2012) this impairment was only found to be significant when compared with the non-verbal IQ control group (*p* <.05; see Figure 2 for sample data). These high levels of spatial error observed in the ASD group were mirrored in high levels of temporal variability. Specifically, the ASD group displayed significantly faster performance times across the trace compared to the non-verbal control group (*p* <.05; see Figure 2 for sample data). Despite apparent similarities between the ASD and receptive language control group, an analysis of prospective control, namely deceleration when approaching corners, successfully distinguished between the ASD and *both* control groups, with significantly shorter phases of corner deceleration being observed in the ASD group (see Figure 3 for sample data). This inability to adequately anticipate the upcoming corner and sufficiently ‘brake’ or decelerate in order to meet the spatial requirements of the task (i.e., stay within the boundaries) implies a specific difficulty with the spatial-temporal control of movement in ASD, which could in turn suggest an underlying problem with perception-action coupling.

### Interceptive skills study (see whyatt and craig, 2013)

In line with qualitative reports (Frith, 2003; Glazebrook et al., 2006), a deconstruction of performance on the M-ABC2 highlighted specific ASD difficulties with catching tasks (Whyatt and Craig, 2012). As previously mentioned, catching is a dynamic action that requires a tight link between one's own movement and the spatial-temporal constraints being imposed by the moving target i.e., the ball. Sufficient levels of movement coupling will ensure the participant synchronizes their movement to the movement of the external target, so they move sufficiently ahead of time to catch the ball. One would therefore expect that initiation times are tailored as a function of the speed of the moving ball toward the target zone, with skilled movement showing a decrease in corrective sub movements and increased successful interception. Apparent difficulties with underlying spatial-temporal control previously demonstrated in the levels of manual dexterity in children with ASD may therefore be further exaggerated when catching a ball, reflecting the persistent results previously found using standardized tests (Whyatt and Craig, 2012).

To further explore potential underlying difficulties with perception-action coupling a controlled catching task was designed, where participants were asked to catch a ball that was rolled down a ramp, in a target zone at the end of the ramp (a task similar to Majsak et al., 1998; see Whyatt and Craig, 2013). Starting and catching areas were fixed for all trials, resulting in a task where all individuals had to move the same distance but adjust how and when they moved as a function of the velocity of the moving ball (adjusted by raising or lowering the ramp between 14 cm (low) and 21 cm (high) settings). To effectively ‘catch’ the ball, participants had to ‘tune into’ or pick up timing information from the movement of the ball to guide their movement to the catching zone so they arrive at the right time. In other words, they have to tailor the temporal characteristics of their movements to the task demands (ball velocity) by coupling perceptual information specifying time to ball arrival to their own actions. Performance in each trial was recorded using Qualisys motion capture infrared cameras, which tracked the movement of the ball (covered in reflective tape) and the hand of the participant (a reflective marker placed on top). Accuracy (number of successful ‘catches’) was measured, and also the ability to modulate initiation time as a function of ball velocity. As before performance was compared between a group of children with ASD and two groups of age-matched controls (receptive language and non-verbal IQ controls).

**Figure 4:**
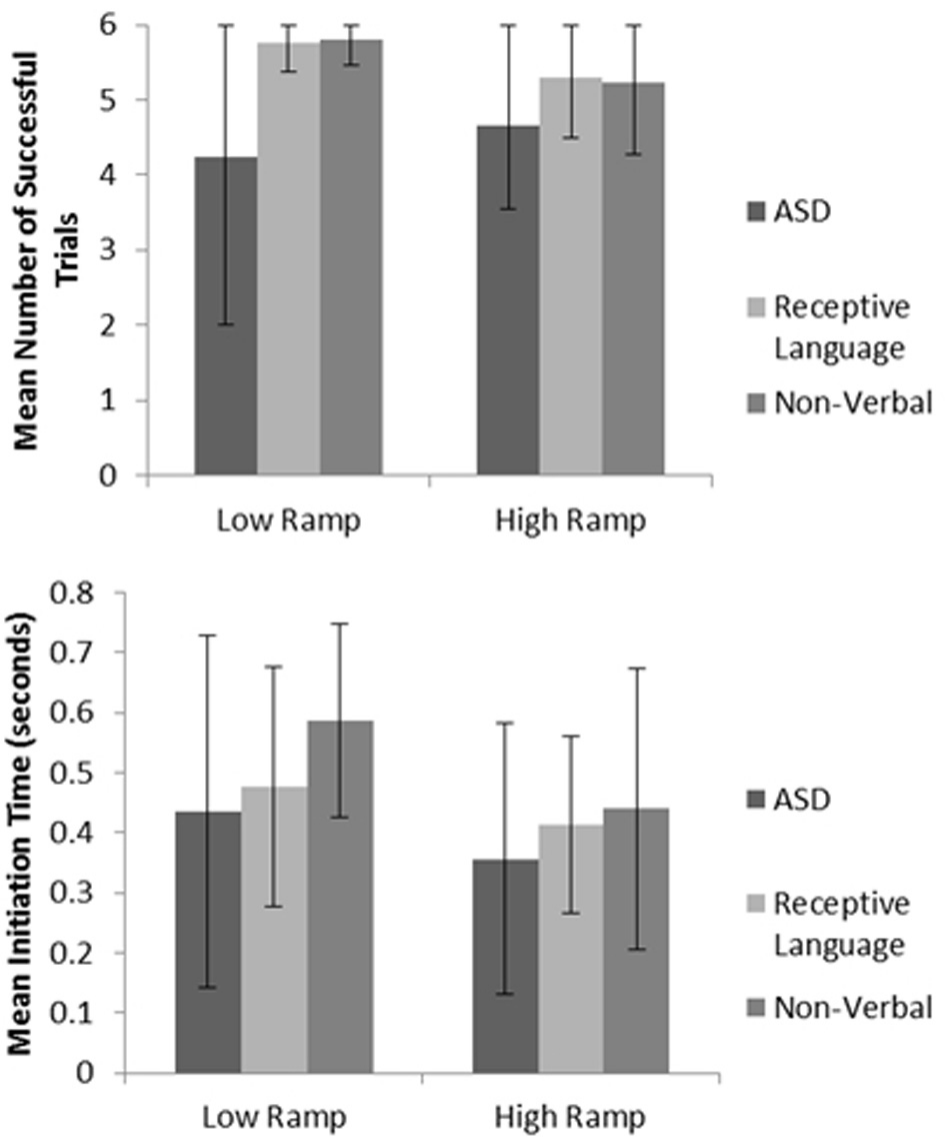
**Example summary graphs for spatial accuracy (measured via number of successful catches), and mean initiation time**. For full data please see Whyatt and Craig (2013).

Mirroring ASD performance found in the manual dexterity study, significantly impaired levels of spatial performance (as measured via successful ‘catches’) were observed when comparing results to those of both the non-verbal and receptive language control groups (*p* < 0.05; see Figure 4 for sample data; also see Whyatt and Craig, 2013). When viewing levels of temporal control, both the ASD and receptive language control groups failed to adequately adapt their initiation times to meet the task demands. For instance, trials using the lower ramp setting, thus lower ball velocity will result in a longer arrival time for the ball. If participants are adequately using sensory information to guide movement, one would therefore expect a longer initiation time. However, the ASD and receptive language groups fail to adapt initiation time to task demands (i.e., ball velocity). In contrast, the non-verbal control group were able to significantly monitor and tailor initiation time to ball velocity (*p* < 0.05), resulting in this group displaying highest levels of overall task success (see Figure 4 for sample data; Whyatt and Craig, 2013). Supporting results from the manual dexterity study, this profile suggests a common underlying difficulty in the ASD and receptive language control group in spatial-temporal control of movement. However, further analysis implies that an ability to guide online necessary temporal modifications to the movement in the receptive language control group compensate for these difficulties with movement initiation (similar to intact corner deceleration profiles shown in the manual dexterity case study). In contrast, the ASD group fails to utilize any sensory information for compensatory strategies, resulting in poor performance.

## Autism: theory of sensory-motor development

Combined with mounting evidence for the presence of significant sensory-motor difficulties in ASD (Fournier et al., 2010), these studies further suggest such lower level problems are a fundamental core symptom of ASD. More specifically this body of work suggests reoccurring prominent difficulties with *manual dexterity* and *ball skills* (e.g., Green et al., 2002, 2009), may be due to underlying variation in the ability to temporally control movement. In particular, the children diagnosed with ASD are found to display an inability to adapt the temporal characteristics of their movement to conform to external spatial constraints. This difficulty emerges as an inability to slow the movement down in complex sections of the manual dexterity task, (e.g., tight turns in corner sections) and an inability adapt to initiation times when intercepting a ball travelling at different speeds to a goal zone. In both cases the children with ASD show higher levels of spatial error than both control groups. Whilst supporting previous studies that suggest an underlying difficulty using visual information to guide movement (Masterson and Biederman, 1983; Gepner and Mestre, 2002; Mari et al., 2003; Minshew et al., 2004; Glazebrook et al., 2006, 2009; Gowen et al., 2008; Dowd et al., 2012), the studies presented above explicitly highlight underlying spatial-temporal control problems which further suggest motor difficulties may be due to a fundamental perception-action coupling deficit.

Although largely taken for granted, perception-action coupling is honed through maturity and experience, and is dependent on the gradual filtering of sensory information to identify sensory invariants to facilitate the establishment of coherent motor control. This filtering or attunement process is dependent on afferent feedback from early exploratory behavior during infancy, which helps teach the infant about the intrinsic properties of the environment, their own abilities, and the relationship between these (Thelen, 1979; Von Hofsten, 2004). These initial explorations are therefore thought to provide the foundations for perception-action coupling, thereby facilitating the progression of meaningful, goal-directed interactions between infants and their surroundings (Von Hofsten, 2004) and the simultaneous decline in early rhythmical exploratory behavior (Thelen, 1979). Reduced levels of goal-directed exploratory behavior during infancy (Pierce and Courchesne, 2001; Ozonoff et al., 2008), the persistence of rhythmical “stereotypies” (Pierce and Courchesne, 2001; Richler et al., 2007), and delayed sensorimotor skill acquisition in ASD (Teitelbaum et al., 1998; Zwaigenbaum et al., 2005), may therefore suggest specific a fundamental problem with perception-action coupling as a consequence of impaired perceptual attunement. Combined, this evidence implies a fundamental difficultly with sensory-motor development in Autism Spectrum Disorders, which may precede later social and cognitive symptoms. Indeed, sensory-motor difficulties may even underline classical symptoms of ASD such as cognition, socialization, and communication (Leary and Hill, 1996; Von Hofsten, 2007; Haswell et al., 2009). Strong links have been repeatedly demonstrated between cognition and motor ability (e.g., Chaix et al., 2007; Dziuk et al., 2007) with both developing in parallel and being mutually dependent (Campos et al., 2000; Von Hofsten, 2007; Rakison and Woodward, 2008; Iverson, 2010). Whilst, a poor internal sense of time in ASD (Boucher, 2001) and variable temporal production may extend to difficulties with the social “dance” such as turn taking and eye contact (Leary and Hill, 1996; Wimpory, 2002). Moreover, growing evidence for substantial links between motor ability and intensity of classical ASD symptoms (Dewey et al., 2007; Freitag et al., 2007; Hilton et al., 2007; Fuentes and Bastian, 2009) further suggest sensory-motor difficulties are potentially a fundamental, core symptom of ASD, which are currently being overlooked.

This inability for children with ASD to use sensory information to guide and time action also suggests that despite similarities between ASD and PD (e.g., Mari et al., 2003; Rinehart et al., 2006a; Hollander et al., 2009) a fundamental difference exists. In particular, PD may be seen as the by-product of a systematic degeneration of the sensory-motor control system, thus reflecting the gradual loss of motor control. In contrast, recent longitudinal and retrospective studies have demonstrated movement problems in children diagnosed with ASD from birth (Teitelbaum et al., 1998; Zwaigenbaum et al., 2005). As such, emerging difficulties with internal temporal control in PD can be successfully minimized by exploiting the pre-established perception-action loop to harness external temporal information (Majsak et al., 1998, 2008). Recent research at the Movement Innovation Lab at Queen's University Belfast has provided additional evidence for the ability of individuals with PD to harness the perception-action loop to maximize movement performance. In particular, this research has demonstrated the use of rich audio and visual temporal ‘cues’ to guide walking performance, balance rehabilitation and reach-grasp movements (Bieñkiewicz, 2011). It is hoped that this research will result in practical implementations to improve quality of life and overall well-being in individuals with PD.

In contrast, movement problems inherent with ASD often encompass both internal and external temporal control issues, thus potentially reflecting a difficulty with the fundamental establishment of coherent and controlled movement. Combined with evidence for persistent sensory-motor difficulties across the spectrum, this suggests the need for early interventions to promote early engaged, exploratory behavior in infants at risk of or with a preliminary diagnosis of ASD. Breaking research has explicitly demonstrated the potential for sensory-motor therapy in ASD (Woo and Leon, 2013), with sensory enrichment (including movement) leading to improved perceptual, social and cognitive functioning in children aged 3–12 years. Sensitivity to the particular sensory preferences and difficulties of an individual, may allow tailored sensory enrichment to facilitate this exploratory process at later stages of development. For instance, advanced motion capture technology can now allow real-time feedback to be presented in relation to positional information. By targeting feedback to the specific sensory preference of the individual, these feedback loops may directly facilitate this exploratory behavior and body mapping by the explicit nature of this perception-action loop.

Moreover, progressive PD includes a battery of ‘non-motor symptoms’, which bear a striking resemblance to classical ASD e.g., pronounced difficulties with ToM, executive functioning tasks, and obsessive compulsive behaviors (Saltzman et al., 2000; Mengelberg and Siegert, 2003; Peron et al., 2009). The dominance of motor symptoms in PD is in stark contrast to the characterization of ASD, in which cognitive and social symptoms are seen as core aspects, with sensory-motor difficulties often referred to as secondary by-products. Substantial evidence for behavioral similarities (Damasio and Maurer, 1978; Vilensky et al., 1981; Mari et al., 2003; Vernazza-Martin et al., 2005; Hollander et al., 2009), coupled with this characterization of PD as a “motor” or “movement” disorder further highlights the importance of sensory-motor problems in ASD, and the need for more objective measurement.

Although the underlying etiology of ASD is still unknown, persistent difficulties with internal timing and preparatory processes imply underlying cerebellar and/or basal ganglia deficits (Paulin, 1993; Graybiel et al., 1994; Courchesne, 1997; Gowen and Miall, 2005). These behavioral manifestations are supported by neuroanatomical research implying reduced basal ganglia and cerebellar activation and neuroanatomical abnormalities in ASD (Allen and Courchesne, 2003; Palmen et al., 2004; Amaral et al., 2008; see also Allen, 2006). The cerebellum is also known to play a critical role in the development and maturation of the sensory integration processes, including visuo-motor integration (Glickstein, 1998). Underlying abnormalities within the cerebellum, commonly present in individuals with ASD (Courchesne et al., 1993; Bauman, 1996; Courchesne, 1997), may therefore emerge as potential problems with sensory integration resulting in a lack of perception-action coupling. This is further supported by evidence for cerebellar hyperactivity in PD, compensating for hypoactivity of the basal ganglia (Yu et al., 2007). This pattern would imply the cerebellum plays a vital role in the exploiting of external sensory temporal information to compensate for underlying difficulties with internal timing, which is moderated by the basal ganglia. This is of particular interest as weak perception-action coupling has previously been shown to be a potential indicator of underlying neurological integrity (Van der Meer et al., 1995; Craig et al., 2000).

However, the question still remains; can these symptoms provide a route of early, non-invasive diagnosis? Initial research implies inherent ASD difficulties with predictive gaze (Von Hofsten et al., 2009), one of the earliest indicators of prospective control (Von Hofsten, 2007), whilst anticipatory deficits are now thought to be a precursor of classical cognitive and social symptoms (Brisson et al., 2011). This is a crucial avenue of future research, as the predictive validity of the social precursors of ASD seems to be questionable prior to 18 months of age (Baranek, 1999). Although not all infants with sensory-motor difficulties will later be formally diagnosed with ASD, the specific nature of sensory-motor difficulties in ASD may be an essential factor. Prominent social and cognitive symptoms may be the measureable, observable product of an underlying difficulty establishing coherent goal-directed, interactive behavior. A new Theory of Sensory-motor control development in ASD may play a critical role in heightening awareness of sensory-motor problems in ASD, whilst providing avenues for preliminary diagnosis. However, for the role of sensory-motor difficulties in ASD to be fully understood it is vital that this particular area of research attracts further support, and a holistic approach is taken. As highlighted, there is an intricate relationship between perception and action, with a need to “move to perceive and perceive to move” (Gibson, 1979), thus neither perception nor motor control can be viewed in isolation. By progressing from abstract tasks, to true, goal-directed tests of sensory-motor control a fuller understanding of the role, and underpinnings of motor deficits may be achieved. Furthermore, examination of motor control through the analysis of kinematic profiles allows an objective assessment of difficulties, removed from product orientated and subjective methods currently adopted in standardized tests and correlational analyses. Given repeated evidence for parallels between ASD and PD, comparing and contrasting kinematic and cognitive performance between these populations may further reveal the relationship between cognitive and motor symptoms. In particular, the disparity in the classification between populations despite strong etiology and behavioral similarities demonstrates the need to explore the complex relationship between motor, cognitive, and social ability.

## Conclusion

In summary, repeated evidence for the presence of significant sensory-motor symptoms across the Autistic Spectrum suggests a traditional cognitive and social view of ASD is short sighted. This work simultaneously highlights both the potential and the limitations of using standardized “norm” based tests commonly used in clinical and research settings. These easy to use standardized tests may provide a gross overview of areas where the motor deficits may reside and can then act as a stepping-stone to unpick sensory-motor difficulties using goal directed tasks with kinematic based analyses. However, if performance was further deconstructed to consider ability at the individual task level additional information may be gained. Moreover the sequential breakdown of performance on a standardized assessment tool (M-ABC2, Henderson and Sugden, 2007) has allowed clear links to be drawn between measurable motor difficulties and underlying kinematic variation. Results also demonstrate the importance of considering both facets of ability when comparing performance across the Autistic spectrum. These results are particularly pertinent given the persistence of significant language delay in ASD, and potential similarities between children with ASD and those with receptive language difficulties (Bartak et al., 1975; Howlin et al., 2000). Such results explicitly highlight the need for this moderating variable to be adequately controlled. Overall it can be seen that motor difficulties are potentially a key component of ASD, rooted in an underlying difficulty with temporal control, due to specific difficulties with perception-action coupling.

### Conflict of interest statement

The authors declare that the research was conducted in the absence of any commercial or financial relationships that could be construed as a potential conflict of interest.
